# Blastic Plasmacytoid Dendritic Cell Neoplasm: A Rare Entity in Clinical Practice

**DOI:** 10.7759/cureus.51860

**Published:** 2024-01-08

**Authors:** Bárbara Oliveira, Carolina Nogueira, Luís Dias, Teresa Ribeiro, Guilherme Gomes

**Affiliations:** 1 Internal Medicine, Hospital de Braga, Braga, PRT; 2 School of Medicine, University of Minho, Braga, PRT; 3 Hematology, Hospital de Braga, Braga, PRT

**Keywords:** rare cancers, cd56+ natural killer (nk) cells, hematological malignancy, bpdcn, non-cutaneous bpdcn

## Abstract

Blastic plasmacytoid dendritic cell neoplasm (BPDCN) is an exceedingly rare and aggressive hematologic malignancy. In the current World Health Organization classification, it is classified among histiocytic/dendritic cell neoplasms. This report describes the case of an 85-year-old female with a complex medical history, including rheumatoid arthritis, who presented with a one-month history of low-grade fever, anorexia, and unexplained weight loss. The diagnosis of BPDCN was confirmed following an immunophenotyping analysis of a bone marrow aspirate. With this report, the authors intend to shed some light on BPDCN's clinical presentation, diagnostic journey, therapeutic approaches, and patient outcomes, and denote the significance of early detection and interdisciplinary collaboration in enhancing patient care.

## Introduction

Blastic plasmacytoid dendritic cell neoplasm (BPDCN) is a rare and aggressive hematologic malignancy that has gained increasing recognition in recent years. It was initially described in 1994 and since then, it has been referred to by various names, including blastic natural killer leukemia/lymphoma, agranular CD4+ natural killer cell leukemia, or agranular CD4+ CD56+ hematodermic neoplasm [1-3]. In the fifth edition of the World Health Organization Classification of Haematolymphoid Tumours, BPDCN is no longer grouped within the class of acute myeloid leukemia-associated precursor neoplasms [4]. Instead, it is now classified under a new category known as 'histiocytic/dendritic cell neoplasms', alongside Langerhans cells and other dendritic cell neoplasms, as well as histiocytic neoplasms.

By presenting this case report, the aim was to highlight the clinical features, diagnostic path, treatment approaches, and patient outcomes of BPDCN, to enrich the existing understanding of this malignancy, while emphasizing the importance of early diagnosis and multidisciplinary collaboration in optimizing patient care.

## Case presentation

The patient, an 85-year-old female with a past history of essential hypertension, foramen ovale, and long-standing erosive rheumatoid arthritis with interstitial lung disease, presented at the emergency department with a one-month history of daily low-grade fever (37.8-38.3ºC), anorexia, and unintended weight loss of approximately 7 kg. In the past two months, she had undergone multiple courses of antibiotics to treat recurrent respiratory infections.

Upon initial assessment, the patient exhibited signs of fatigue and debilitation. Examination revealed pericentimetric, mobile, and tender-to-touch right axillary lymphadenopathy. Abdominal examination did not reveal any palpable masses or organ enlargement, and no skin lesions were observed.

**Figure 1:**
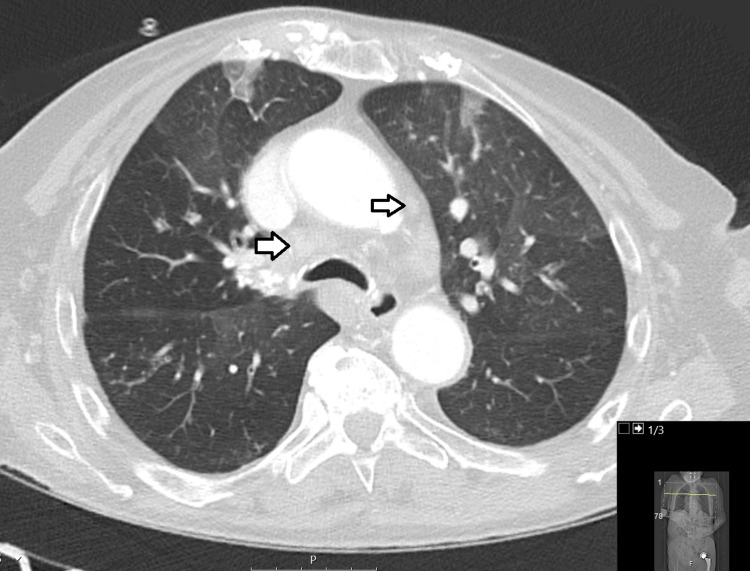
CT scan of the chest showing hilar lymphadenopathies.

Laboratory results revealed a hemoglobin level of 8.9 g/dL (reference range: 11.9 to 15.6 g/dL), white blood cell count of 3.5x10^3^/µL (reference range: 4 to 11x10^3^/µL), and platelet count of 55x10^3^/µL (reference range: 150 to 400x103/µL). Peripheral blood smear revealed the presence of medium-sized blasts with a monocytoid-like morphology, single nucleoli, and abundant cytoplasm with occasional vacuoles. CT scan of the chest and abdomen showed hilar and mesenteric lymph node enlargement, with no other relevant alterations noted (Figure 1).

Peripheral blood immunophenotyping revealed the presence of T and B lymphopenia and an increase in plasmablasts. A bone marrow aspirate was performed, and the immunophenotyping analysis revealed the presence of a population of cells with positivity to CD56 and CD123, leading to the diagnosis of BPDCN.

A hematology consultation was sought. Considering the patient's overall poor condition and limited treatment options, she was referred to palliative care. Tragically, she succumbed to her condition one week later.

## Discussion

BPDCN typically occurs in elderly patients, with a mean age at diagnosis of 60-70 years old, but it can present at any age [5]. It is more prevalent in males, with a male-to-female ratio of approximately 3:1 [6]. It can present as an isolated neoplasm or in conjunction with other hematological malignancies such as chronic myelomonocytic leukemia (CML) or myelodysplastic syndrome (MDS).

It typically involves multiple sites, with the skin being the most frequently affected (60-100%), followed by peripheral blood and bone marrow (60-90%), and lymph nodes (40-50%). In fact, the skin is often the initial site of involvement in most patients, prompting them to seek medical attention. Skin lesions can manifest as nodules or bruise-like lesions, varying in number, shape, and size [7]. However, there have been reports of patients presenting with a leukemia-like picture despite the absence of cutaneous disease [8]. When bone marrow is involved, several peripheral blood abnormalities often occur, including thrombocytopenia (78%), anemia (65%), and neutropenia (34%). While leukocytosis is relatively rare, the presence of circulating blasts is a more frequent finding. Lymphadenopathy, splenomegaly, and hepatomegaly are observed in approximately 56%, 44%, and 42% of patients, respectively [7]. In addition, reports have documented involvement in various other sites, such as the tonsils, soft tissues, paranasal cavities, lungs, eyes, and the central nervous system [8].

The diagnosis of BPDCN relies on a biopsy and/or morphological evaluation of the affected tissue, coupled with immunophenotypic testing through either flow cytometry or immunohistochemistry. BPDCN is defined by distinctive histopathological characteristics. When examined under the microscope, BPDCN cells typically exhibit a widespread infiltration of medium-sized blasts, characterized by irregular nuclei, fine chromatin, and the presence of single to multiple nucleoli. Giemsa staining reveals a telltale narrow, grayish-blue, and agranular cytoplasm rim in affected cells. Small vacuoles and/or pseudopodia can also be seen in some patients. More rarely, blast cells can appear with a dominant lymphoid-like morphology (18% of the cases) or a monocytoid-like morphology (6% of the cases) [9]. Additionally, assessing the proliferation rate through the Ki-67 index indicates that the neoplastic cells can exhibit varying levels of proliferation, often falling within a range of 20-80% [6].

BPDCN diagnosis through immunohistochemical analysis relies on the presence of specific markers, notably CD4, CD56, and CD123, and the identification of other markers with greater specificity for plasmacytoid dendritic cells, such as TCF4, TCL1, CD303, or SPIB. Simultaneously, the absence of markers associated with lymphoid, natural killer, and myeloid lineages is essential for diagnostic confirmation [10].

Managing BPDCN presents significant challenges due to its rarity and aggressive nature. Historically, treatment strategies included localized surgical or radiation therapy for skin-confined cases. In cases of more extensive disease involvement, multi-agent cytotoxic chemotherapy regimens were employed, with the protocol for acute lymphoblastic leukemia showing superior effectiveness compared to acute myeloid leukemia protocols [6].

In recent years, targeted therapies directed at CD123 have gained attention and demonstrated promising results in BPDCN treatment. However, relapse rates remain high, and a cure is typically not achieved without undergoing a hematopoietic stem cell transplant. A recent study has introduced the use of a cytotoxic chemotherapy regimen based on hyperfractionated cyclophosphamide, vincristine, adriamycin, and dexamethasone, which has shown high rates of complete remission [11]. Further research is necessary to determine the optimal combination of targeted therapies and cytotoxic treatments that can lead to durable remission.

## Conclusions

BPDCN remains a challenge in the realm of hematologic malignancies. Its rarity and aggressive behavior require a multidisciplinary approach. While advances in understanding this enigmatic disease are being made, challenges such as high relapse rates and the elusive pursuit of durable remission persist.

As we continue to unravel the complexities of BPDCN, the collaborative efforts of clinicians and researchers are of utter importance. With further research, the development of innovative therapies, and the relentless pursuit of tailored treatment strategies, we strive to improve the prognosis of individuals affected by this rare and aggressive hematologic malignancy.
